# Authors’ Reply: “The Need for a Bleed Type–Specific Annual Bleeding Rate in Hemophilia Studies”

**DOI:** 10.2196/54756

**Published:** 2024-03-13

**Authors:** Limin Wang, Shimeng Liu, Shan Jiang, Chaofan Li, Liyong Lu, Yunhai Fang, Shunping Li

**Affiliations:** 1 Centre for Health Management and Policy Research School of Public Health, Cheeloo College of Medicine Shandong University Jinan China; 2 NHC Key Laboratory of Health Economics and Policy Research Shandong University Jinan China; 3 Centre for Health Preference Research Shandong University Jinan China; 4 School of Public Health Fudan University Shanghai China; 5 NHC Key Laboratory of Health Technology Assessment Fudan University Shanghai China; 6 Macquarie University Centre for the Health Economy Macquarie Business School and Australian Institute of Health Innovation Macquarie University Sydney Australia; 7 Shandong Hemophilia Treatment Center Shandong Blood Center Jinan China

**Keywords:** benefit-risk assessment, discrete choice experiment, hemophilia A, patient preference, prophylactic treatment

We appreciate Huang’s [1] thoughtful consideration of our article [2]. Their suggestion to refine the attribute of “annual bleeding rate” in our discrete choice experiment (DCE) by distinguishing between spontaneous versus injury-induced bleeding, and further categorizing bleeds as joint or mild, is acknowledged with appreciation. In the formulation of our DCE questionnaire, considerable attention was devoted to addressing this aspect. However, the integration of a comprehensive spectrum of bleed types into the DCE was met with challenges, particularly within the context of the current hemophilia A scenario in China and the specific focus of our investigation.

Our study focused on adult patients with hemophilia. With the notable increase in the adoption of low-dose prophylactic treatment and advancements in diagnostic and therapeutic capabilities, there has been a significant rise in pediatric patients benefiting from prophylaxis with coagulation factor [3]. However, it is important to highlight that the current percentage of adult patients undergoing prophylaxis with coagulation factor remains relatively low [4]. In our DCE, the number of annual bleeds attribute was operationalized as spontaneous bleeding events. The decision not to delve into further details of bleeding types in the DCE was primarily influenced by the reasons listed below.

First, patients have limited ability to assess their own bleeding, and misrecording or missing records often occur [5]. Unlike during a clinical trial, it is challenging for them to obtain detailed descriptions of all types of bleeds per year via questionnaire. Second, while adult patients with hemophilia in developed countries engage in physical activities at rates comparable to the general population, the situation in China is markedly different [6]. With age, patients often exhibit varying degrees of joint damage or deformities, particularly in the knees, ankles, and elbows, resulting in a generally reduced activity level [7]. Consequently, the incidence of activity- or injury-induced bleeding is relatively low. Moreover, a significant portion of adult patients in China may already be experiencing joint deformities or at a stage requiring joint replacement [4], rendering the notion of “target joints” less relevant.

In the DCE context, it is crucial that the chosen attribute and levels align with practical conditions, ensuring ease of investigation and data collection from the participants. Therefore, for this study, we selected the number of annual bleeds (spontaneous bleeding) as the attribute. Our DCE design, particularly in determining attributes and levels, entailed multiple rounds of consultations and interviews with clinical experts, including hemophilia specialists from the Shandong Hemophilia Treatment Center (the core center of the Hemophilia Treatment Center Collaborative Network of China and the World Federation of Hemophilia). These consultations were instrumental in providing valuable professional guidance.

We believe that these additional details provide a comprehensive background of our DCE design. Furthermore, our aspiration is to enhance access to prophylaxis with coagulation factor for adult patients in China, accompanied by a commitment to maintain precise bleeding records. This commitment would not only benefit our study but also pave the way for future research to refine the measurement of annual bleeding frequency by incorporating additional indicators like bleeding types.

## Abbreviations

DCE: discrete choice experiment
